# Cognitive Deterioration and Quality of Life in Patients with Schizophrenia: A Single Institution Experience

**DOI:** 10.7759/cureus.6772

**Published:** 2020-01-25

**Authors:** Jorge Avila, Larissa Villacrés, Diego Rosado, Evelyn Loor

**Affiliations:** 1 Internal Medicine, Kennedy Hospital, Guayaquil, ECU; 2 Medicine, Espíritu Santo University, Guayaquil, ECU; 3 General Medicine, Espíritu Santo University, Guayaquil, ECU

**Keywords:** schizophrenia, cognitive impairment, quality of life

## Abstract

The present article aimed to analyze and describe the relationship between cognitive impairment and the perception of quality of life. The study was carried out in the psychiatric hospital within the Ecuadorian “Instituto de Neurociencias,” with 50 patients, both men and women, between the ages of 18 and 65 years. An instrument for the screening of cognitive impairment in psychiatric patients, a questionnaire that measures quality of life, and a sociodemographic questionnaire were applied individually. Data collection took between 30 and 40 minutes per person. The results obtained allowed to verify the relationship between cognitive deterioration and quality of life. First, it was shown that while there was greater cognitive impairment, the perception of quality of life was low and vice versa. It was found that the areas of verbal learning, processing speed, and working memory are those that present greater difficulty for patients with this disorder.

## Introduction

Within the study of psychiatric and psychological disorders, one of the pathologies that has had a great transcendence in this area is schizophrenia. The mind and the brain are so fundamental to the human being that any disturbance in their functioning can cause consequences in most areas where a subject develops. Approximately 1% of the population suffers from this disorder, but the risk of suffering from it is 0.2%-2% in the general population [1]. Schizophrenia is a problem that should be studied in greater depth since, in developed countries, "it occupies the fifth place in the list of disorders associated with severe disability" [2] while the World Health Organization (WHO) reports that 50% of people with this diagnosis cannot access adequate treatment and that 90% of people with schizophrenia live in underdeveloped countries [1].

In Ecuador, there are several studies focused on mental health, but there are no actual studies on schizophrenia. Most of them focus on quantifying and maintaining statistical data on mental pathologies, but there is no interest in investigating other aspects of these patients or even the consequences of such diagnosis, especially cognitive impairment and life quality.

It is suggested that patients with schizophrenia have cognitive impairment, with a greater deficit in attention, memory, and executive functions than the general population. The levels of deterioration vary greatly between patients but the constant is the presence of deficit. This deficit seems independent of circumstances beyond the disease such as medication or institutionalization [3].

Negative symptoms, psychiatric comorbidity, and the side-effects of drug treatment are factors that negatively influence the life quality (LQ) of people with schizophrenia. “Regarding the type of drug, atypical antipsychotics are chosen, rather than the typical ones, because they have a greater contribution to the approach of positive and negative symptoms, which contributes to an increase in LQ” [4]. Adherence with treatment and the cost of constant medical-psychological care for people with low income are factors that can decrease their LQ and, even when there are no resources, lead to a relapse of symptoms.

This article states as a hypothesis the fact that there is a considerable relationship between cognitive impairment and life quality in patients suffering from the disease. There are researches about cognitive impairment or quality of life in these cases but not one that encompasses the two fields. For this reason, 50 patients diagnosed with schizophrenia (paranoid, residual, and catatonic, among others), both female and male, between 18 and 65 years of age, who are in the psychiatric hospital of the Ecuadorian Instituto de Neurociencias (Institute of Neurosciences) were chosen as the universe for this article.

The objective of this research was to contribute relevant data on the level of global cognitive impairment in schizophrenic patients and correlate it to aspects related to their life quality. The methodology used focuses on the use of validated scales and tests to obtain and gather reliable data on the subject to be evaluated. Among these evaluation instruments, there is a sociodemographic questionnaire, the Screening of Cognitive Deterioration in Psychiatry (SCIP), and the World Health Organization Quality of Life (WHOQOL).

Early diagnosis, pharmacological and psychotherapeutic monitoring, relapse prevention, and family, social, and work support could reduce cognitive impairment and increase life quality in patients with schizophrenia. In turn, this would allow fostering interpersonal relationships, self-reliance, and self-confidence in these patients and thus allow a greater contribution to their personal and their country´s development.

## Materials and methods

This is a cross-sectional, quantitative, correlational, and descriptive study. The population of the study involved 50 schizophrenic patients who are hospitalized in the psychiatric hospital of the Ecuadorian Instituto de Neurociencias (Institute of Neurosciences) between the months of September and November 2016. The project was presented to Espíritu Santo University in Ecuador for its evaluation by an ethics committee, and after its approval, it was sent to the Neuroscience Institute of Guayaquil for permission. Data collection was carried out with the prior authorization of the research and teaching directors of the mentioned institute, and informed consent was granted by the participating patients. Patients with the diagnosis of schizophrenia or one of its subtypes (paranoid, residual, catatonic, and so on) were chosen. Of these chosen patients, 50 participants were selected through non-probabilistic sampling (25 women and 25 men); their ages were between 18 and 65 years old. Three instruments were applied to the participants: a questionnaire (age, sex, education, employment status, diagnosis time, number of admissions, and so on), a test that measures cognitive impairment in psychiatric patients (SCIP-S), and the Instrument of Evaluation of Quality of Life of the World Health Organization (WHOQO-BREF).

The SCIP-S is a cognitive screening test specifically developed to detect the main cognitive deficits that people with some type of mental illness develop [5]. It consists of five sub-tests, which measure memory, attention, processing speed, and executive function. The results generate a cognitive profile of the patient in addition to an overall score, which is the one used to correlate the data.

The WHOQOL-BREF is an instrument designed for the evaluation of the perception of life quality, within the cultural context and value system in which people live, together with their objectives, goals, and interests. The short version was used because for the population evaluated, the instruments to be used should be of short duration.

Data were collected anonymously, signing an informed consent form. The objective of the investigation, the content of the instruments, and the application were explained. The total time per person was approximately 30 to 40 minutes. After data collection, statistical descriptive tables on cognitive impairment and life quality were developed in patients diagnosed with schizophrenia.

## Results

Fifty participants were studied (25 women and 25 men). The average age was 41.5 years, with the youngest patient being 18 and the oldest being 65. Regarding the type of schizophrenia presented, 22 (44%) were classified as paranoid schizophrenia, 14 (28%) as undifferentiated, eight (16%) had a diagnosis of residual schizophrenia, five (10%) as hebephrenic, and one (2%) patient was hospitalized with the diagnosis of unspecified schizophrenia (Table 1).

**Table 1:** Characteristics of patients diagnosed with schizophrenia included in this study

Variables	Levels	n	Percentage
Sex	Male	25	50%
	Female	25	50%
Type of schizophrenia	Paranoid	22	44%
	Undifferentiated	14	28%
	Residual	8	16%
	Hebephrenic	5	10%
	Unspecified	1	2%
Cognitive qualities score	Very low	15	30%
	Low	12	24%
	Medium low	5	10%
	Medium	16	32%
	Medium high	2	4%
	High	0	0%
Schooling level	Primary schooling or inferior education	22	44%
	Secondary schooling or superior education	28	56%
Life quality perception	Low	23	46%
	Medium	20	40%
	High	7	14%
Presence of a primary caregiver	Yes	12	24%
	No	38	76%

Regarding the overall result of cognitive impairment in the patients evaluated, the diagnosis of impairment indicates that 30% (n = 15) of those evaluated obtained a “very low” rating on their cognitive qualities; 24% (n = 12) obtained the “low” diagnosis; 10% (n = 5) the diagnosis of "medium low"; 32% (n = 16) obtained the diagnosis of "medium"; 4% (n = 2) the diagnosis of "medium high," and 0%, meaning 0 evaluated, obtained the diagnosis of "high."

Concerning ​​cognitive impairment, the results show that processing speed (32%, n = 16) was the most affected cognitive area while the other areas' results were deferred verbal learning (28%, n = 14), verbal fluency (22%, n = 11), immediate verbal learning (20%, n = 10), and working memory (12%, n = 6). The least affected area was immediate verbal learning (Table 2), presenting the greatest amount of success in the tests.

**Table 2:** Areas of cognitive evaluation and their scores

Diagnosis	Working memory	Verbal fluency	Immediate verbal learning	Deferred verbal learning	Processing speed
Very low	12%	22%	20%	28%	32%
Low	36%	24%	28%	32%	38%
Medium low	18%	14%	12%	10%	10%
Medium	22%	26%	22%	30%	14%
Medium high	12%	14%	16%	0	6%
High	0	0	2%	0	0

**Figure 1:**
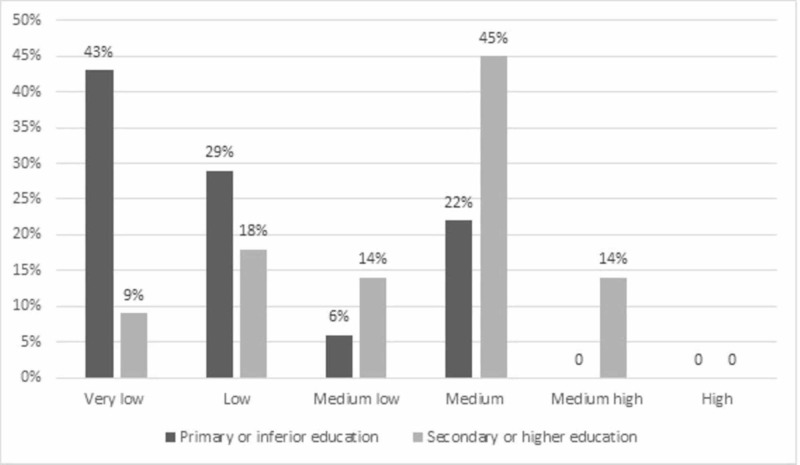
Bar graph of the relationship between schooling and cognitive impairment

Regarding the level of schooling and its relationship with cognitive impairment, it can be observed that the majority of patients who completed primary studies or any type of education lower than primary schooling obtained a “very low” score (43%), the scores of “medium high ”(0%) and “high ”(0%) were not obtained. Whereas, of the patients who completed high school or higher studies, they obtained mostly 45%, a “medium” score; the “high” score (0%) was not obtained (Figure 1).

**Figure 2:**
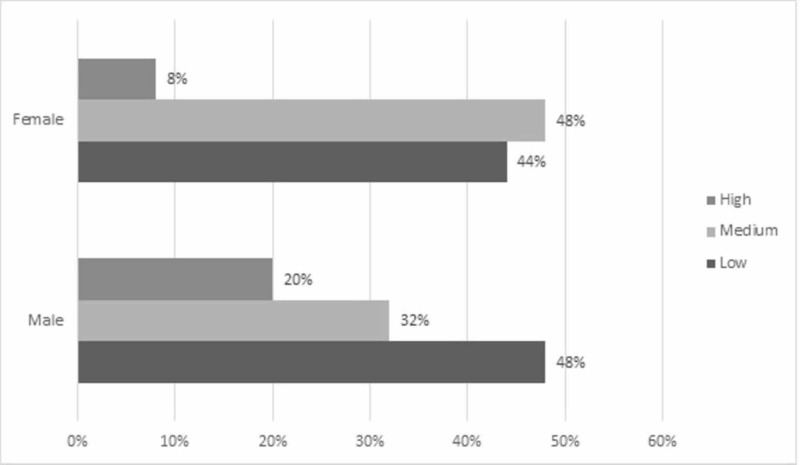
Bar graph of the percentage of perception of life quality according to sex

With respect to the perception of quality of life in the male patients evaluated, 12 (48%) considered having a low life quality, eight (32%) considered an average life quality, and five (20%) answered high life quality. While in females, 11 (44%) responded as low life quality, 12 (48%) considered an average life quality, and two (8%) considered a high life quality. It can be concluded that it is possible to observe that a low life quality predominates among the male evaluated, while in women, a medium life quality does so (Figure 2).

The relationship between the presence of a primary caregiver or the absence of one regarding life quality indicates that 100% of patients who maintain a "high" perception of their quality of life present a primary caregiver. On the contrary, 91% of patients with a "low" quality of life do not have a primary caregiver.

Concerning the relationship between cognitive impairment and quality of life, those who obtained "very low" in cognitive testing, mostly have a "very low" life quality (79%). While those who obtained "medium high" in the evaluation of cognitive impairment, zero presented a "low" perception of life quality, 33% a "medium life quality," and 67%, a "high life quality."

## Discussion

Through this study, important relationships between cognitive impairment and life quality were found. First, cognitive impairment has a significant influence on life quality. As mentioned, as more cognitive deterioration occurs, life quality will decrease and vice versa. Second, it was observed that verbal learning, processing speed, and working memory were the most affected cognitive areas. Finally, the importance of the presence of a primary caregiver for schizophrenic patients was determined, as it is one of the critical points that affect their quality of life.

Matsui et al. studied the cognitive function related to quality of life in patients with schizophrenia, and their results are very similar to ours. These results demonstrated that patients with schizophrenia have deficits in executive function, memory and learning, and social knowledge. Thus, in patients with schizophrenia, deficits in social knowledge appear to be associated with the current quality of life in these patients and specifically with the capacity for empathy and social initiative [6].

Caron et al. described that the increased well-being and quality of life in people with schizophrenia were related to higher levels of education [7]. This finding is consistent with ours.

Finally, Caqueo-Urízar et al. presented a bidirectional association between the presence of a caregiver in the quality of life of schizophrenic patients, showing that good quality of life in the caregiver affects the patient positively and vice versa. The study also indicated that the involvement of the family in the patients' lives has a positive impact on their quality of life [8].

## Conclusions

According to our study, quality of life in schizophrenic patients is inversely proportional to the degree of cognitive deterioration. The factors that affect the quality of life of these patients positively include higher levels of education, the presence of a caregiver, and low levels of cognitive impairment. We recommend that patients receive care from a family member in order to slow the deterioration of quality of life and cognitive impairment, as it has been shown that it affects the patient's development positively. However, more studies are needed to correlate this disease with other characteristics.
